# A Hybrid PCA-CART-MARS-Based Prognostic Approach of the Remaining Useful Life for Aircraft Engines

**DOI:** 10.3390/s150307062

**Published:** 2015-03-23

**Authors:** Fernando Sánchez Lasheras, Paulino José García Nieto, Francisco Javier de Cos Juez, Ricardo Mayo Bayón, Victor Manuel González Suárez

**Affiliations:** 1 Department of Construction and Manufacturing Engineering, University of Oviedo, Gijón 33204, Spain; 2 Department of Mathematics, University of Oviedo, Oviedo 33007, Spain; E-Mail: lato@orion.ciencias.uniovi.es; 3 Department of Mining Engineering and Exploitation, University of Oviedo, Oviedo 33004, Spain; E-Mail: fjcos@uniovi.es; 4 Department of Electrical Engineering, University of Oviedo, Gijón 33204, Spain; E-Mails: rmayo@uniovi.es (R.M.B.); vmsuarez@uniovi.es (V.M.G.S.)

**Keywords:** prognostics, aircraft engine, remaining useful life, principal component analysis (PCA), dendrogram, classification and regression trees (CART), multivariate adaptive regression splines (MARS)

## Abstract

Prognostics is an engineering discipline that predicts the future health of a system. In this research work, a data-driven approach for prognostics is proposed. Indeed, the present paper describes a data-driven hybrid model for the successful prediction of the remaining useful life of aircraft engines. The approach combines the multivariate adaptive regression splines (MARS) technique with the principal component analysis (PCA), dendrograms and classification and regression trees (CARTs). Elements extracted from sensor signals are used to train this hybrid model, representing different levels of health for aircraft engines. In this way, this hybrid algorithm is used to predict the trends of these elements. Based on this fitting, one can determine the future health state of a system and estimate its remaining useful life (RUL) with accuracy. To evaluate the proposed approach, a test was carried out using aircraft engine signals collected from physical sensors (temperature, pressure, speed, fuel flow, *etc.*). Simulation results show that the PCA-CART-MARS-based approach can forecast faults long before they occur and can predict the RUL. The proposed hybrid model presents as its main advantage the fact that it does not require information about the previous operation states of the input variables of the engine. The performance of this model was compared with those obtained by other benchmark models (multivariate linear regression and artificial neural networks) also applied in recent years for the modeling of remaining useful life. Therefore, the PCA-CART-MARS-based approach is very promising in the field of prognostics of the RUL for aircraft engines.

## 1. Introduction

In this section an introduction to prognostics and health management (PHM) is performed. Also, the aircraft sensors are briefly described and the functioning of the aircraft engine is explained. PHM is now an engineering discipline with a growing importance. PHM combines [1] detection and analysis of environmental, operational and performance-related parameters to assess the health of a product and predict its remaining useful life (RUL). The current state of the art of the PHM [2] presents three main research lines:

- *Data-driven approach*: The data-driven models for the prediction of the health or RUL of any system uses machine learning and pattern recognition techniques [3,4,5]. The main characteristic of this kind of models is that they do not require any previous knowledge about the device under analysis as the prediction is made according to the available information without taking into account the operating principles of the system.
- *Model-based approach*: in this approach the prognostic models are based on an understanding of the physical process and interrelationships among the different components and subsystems of the device [6]. The model-based-approach not only includes system modeling but would also apply physics of failure modeling [1] or any similar methodology.
- *Hybrid approaches*: These kinds of models try to make the most of both data-driven and model based approaches [1]. A hybrid model combines both data-driven methodologies with the knowledge of the device under study. Please do not confuse PHM hybrid approach with hybrid data-driven models, which are models that combine different pattern-recognition and machine learning models in order to predict either the RUL or the status of the system under study.

Although the two traditional approaches to PHM have been model-based or data driven, some of the latest research in the PHM discipline combines the strength of the data-driven prognostic methods with the physical models of the system under study, achieving remarkable results [7] in spite of this, data driven approaches like the one presented in this research are also shown to be performing well [8].

The methodology presented in this paper can be considered as data-driven and proposes a model that combines well-known pattern recognition and machine learning methodologies in a hybrid model that is trained in order to be able to predict the RUL of the aircraft engine under study taking into account the current status of some of its variables, but without requiring information about the previous status of the variables of the engine. In other words, the concept of hybrid algorithm does not refer to an algorithm that combines both data-driven methodologies with the understanding of the system under study but rather that it refers to a hybrid algorithm with the meaning that it is usually employed in the field of machine learning and pattern recognition [9,10]. The model proposed hybridizes the following techniques: Principal Component Analysis (PCA), Dendrogram, Regression Trees and Multivariate Adaptive Regression Splines (MARS). The advantages of the proposed algorithm are twofold; on the one hand a reduced number of variables is employed for its training, which makes the training of the final models more efficient and their results easier to replicate, while on the other hand the final MARS models that are trained are not for the complete state of space but for a subset which increases the accuracy of predictions over an individual method. Although there have been previous papers that employ data-driven models based on hybrid algorithms [2,11], the methodology proposed in this paper is new and performs well when compared with previous models used with the same database.

In the present research, the concept of RUL [12] must be understood to be the number of time units remaining that the equipment has before it reaches its limit of operative safety. In recent years a vast number of articles related to different approaches to the concept of remaining useful life based on statistical concepts have been written. In general, the remaining useful life of any device can be considered as a random variable [12].

### The Aircraft Engine Sensors

Aircraft engine monitoring systems are used to check the health of the aircraft engines in order to avoid costly repairs by means of preventive maintenance. Engine monitoring systems involve using sensors placed in various locations in an aircraft engine to gather information about its performance. The sensors provide real-time information to pilots on the operation of the engines and also capture data for analysis of the performance of the engine over time. The information obtained provides information about the engine status. The main kinds of sensors present in an aircraft engine are the following:

- *Temperature sensors*: These provide output readings proportional to temperature. The measurement of temperature can be accomplished using thermocouple or resistance temperature device (RTD) techniques depending upon system interface, temperature, or accuracy considerations.
- *Pressure sensors*: Currently the most common pressure sensors are those formed by two separate absolute pressure-sensing capsules. One senses system pressure and the other atmospheric pressure. In these devices, the output signal is the difference between the signals of both sensors.
- *Sensors of speed (rpm)*: These kinds of sensors are usually installed directly on the low-pressure compressor rotor in order to determine the revolutions at the low-pressure section. They are also installed in the casing of driving mechanisms, as this is the most suitable place for the measurement of the speed of the high-pressure compressor section.
- *Sensors of fuel flow*: These are usually vane sensors located downstream behind the fuel filter and the pump measuring the amount of fuel going to the engine. The importance of the fuel flow sensor is remarkable as it can immediately detect any engine abnormality involving an increase in fuel consumption.

**Figure 1:**
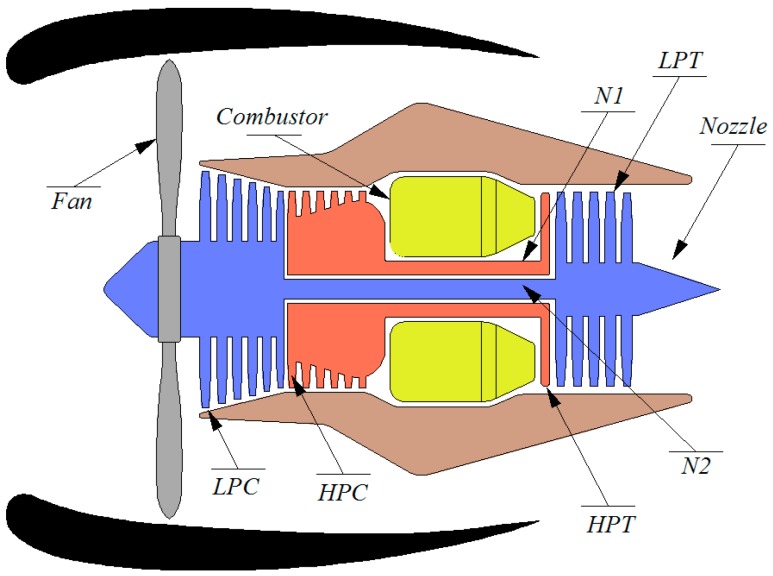
Simplified diagram of the engine simulated [13] (LPC: Low pressure compressor, HPC: High pressure compressor, LPT: Low pressure turbine, HPT: High pressure turbine, N1: Turbine axis and N2: Turbine shaft).

The diagram in Figure 1 shows the main elements of the engine model proposed in the present research. Air gets into the low pressure compressor through the fan passing afterwards to the high pressure compressor. The air is then heated in the combustor where it is mixed with fuel and ignited. Combustion of the fuel increases the HPC discharge air velocity to drive the high and low pressure turbines. This engine model is based on a low frequency, transient, performance model of a high-pressure ratio, dual-spool, low by-pass, variable cycle, turbofan engine with a digital controller. The controller update rate is 50 Hz, and the component level model balances the mass/energy equations of the system at a rate of 2500 Hz. These capabilities allow the user to verify the performance of the control algorithms and its validation in a generic engine model. The MAPSS program has versions for both civil and military application. A complete explanation of the full C-MAPSS subsystem hierarchy can be found in the C-MAPSS User’s Guide [13]. The military version is able to perform realistic simulations within the standard Full Authority Digital Engine Controllers (FADEC) [14] and it will be used in the present research work.

## 2. Materials and Methods

The materials and methods section explains the variables included in the database used for this research and how they were obtained. This section also describes the hybrid algorithm that is the original contribution of this research and all the statistical procedures that are part of it.

### 2.1. The Database

The present research uses data corresponding to an aircraft engine of 90,000 lb thrust class. The data includes working conditions with heights from sea-level conditions up to heights of 40,000 feet and temperatures ranging from −51 °C to 39 °C. This data was obtained through a software application known as Modular Aero-Propulsion System Simulation (MAPSS). This is a simulation environment for aeronautical turbines which allows access to a range of monitoring parameters controlling the operating state of the system through a graphical user interface. This provides the user with a simulation environment which is able to develop advanced algorithms for control and diagnosis that can be tested on a generic aircraft engine simulator. MAPSS is able to generate linear state space models from which the user can create linear controls. Furthermore, MAPSS is able to perform simulations of transient states. These capabilities allow the user to verify the performance of the control algorithms and its validation in a generic engine model [14]. The MAPSS program has versions for both civil and military application. The military version is able to perform realistic simulations within the Full Authority Digital Engine Controllers (FADEC) [14] standard and will be used for the present research.

The database contains input parameters that simulate the effects of faults and deterioration in any of the rotating components of the engine (fan, low pressure compressor or LPC, high pressure compressor or HPC, high pressure turbine or HPT, and low pressure turbine or LPT). The data belongs to a fleet of similar aircraft with the same engines. Each engine starts with a different health status, but initially all are within the operational range and the data set convers from that moment to the engine failure. Each record of the engine state is formed by a set of 24 variables. Three of these are operational settings, while the other 21 represent values for measurements of engine performance which are contaminated with noise. Also, the remaining useful life of the engines for each record is known. Please note that the length of the RUL is a theoretical time unit that would be considered in either hours or cycles. This information has been introduced in the manuscript, in the section that describes the database. A complete list of variables is presented in Table 1. This database will be used for the training and validation of the hybrid model proposed in this paper. All the engines considered in this study had the same failure mode, namely, HPC degradation. To simulate HPC degradation, HPC flow and efficiency modifiers were used [12].

**Table 1:** MAPSS input variables of the simulated engine to simulate the remaining useful life (RUL).

Input Variables	Symbol	Sensor
Total temperature at fan inlet (°R)	T2	Sensor.Measurement1
Total temperature at LPC outlet (°R)	T24	Sensor.Measurement2
Total temperature at HPC outlet (°R)	T30	Sensor.Measurement3
Total temperature at LPT outlet (°R)	T50	Sensor.Measurement4
Pressure at fan inlet (psia)	P2	Sensor.Measurement5
Total pressure in bypass-duct (psia)	P15	Sensor.Measurement6
Total pressure at HPC outlet (psia)	P30	Sensor.Measurement7
Physical fan speed (rpm)	Nf	Sensor.Measurement8
Physical core speed (rpm)	Nc	Sensor.Measurement9
Engine pressure ratio (P50/P2)	Epr	Sensor.Measurement10
Static pressure at HPC outlet (psia)	Ps30	Sensor.Measurement11
Ratio of fuel flow to Ps30 (pps/psi)	phi	Sensor.Measurement12
Corrected fan speed (rpm)	NRf	Sensor.Measurement13
Corrected core speed (rpm)	NRc	Sensor.Measurement14
Bypass ratio	BPR	Sensor.Measurement15
Burner fuel-air ratio	farB	Sensor.Measurement16
Bleed enthalpy	htBleed	Sensor.Measurement17
Demanded fan speed (rpm)	Nf_dmd	Sensor.Measurement18
Demanded corrected fan speed (rpm)	PCNfR_dmd	Sensor.Measurement19
HPT coolant bleed (lbm/s)	W31	Sensor.Measurement20
LPT coolant bleed (lbm/s)	W32	Sensor.Measurement21

### 2.2. The Proposed Algorithm

**Figure 2:**
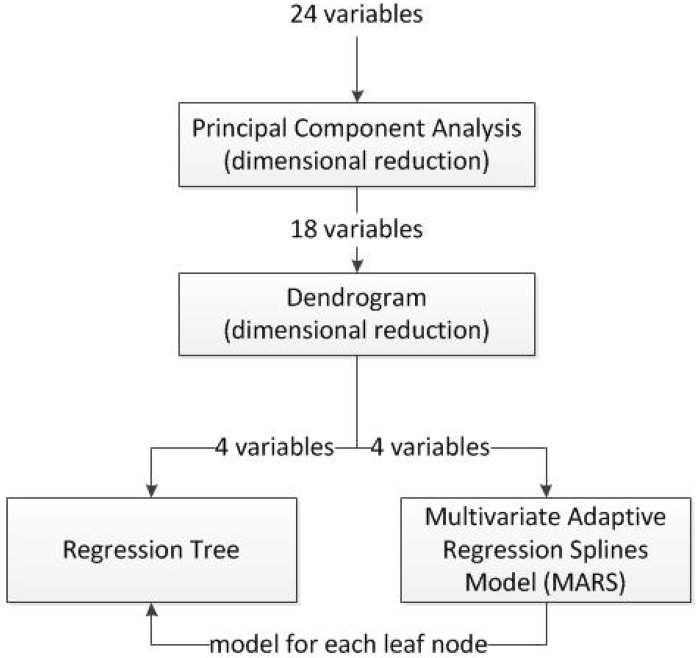
Flowchart of the proposed algorithm.

The model proposed in this research is a hybrid model. In recent years, hybrid methods in machine learning have attracted a great deal of attention from the scientific community. There has been an increasing tendency to use of hybrid models due to the good performance which these kinds of models have given, not only in the forecast of RUL [15,16] but also in many other different applications [17,18]. In other words, it may be observed from the comparison between methods and hybridizations that the performance of the hybrid method is superior [19,20]. The steps followed in the proposed hybrid algorithm are shown in Figure 2. The steps of this algorithm are as follows:

- *Dimensional reduction by means of Principal Components Analysis (PCA)*: In the first stage, a dimensional reduction by means of PCA was performed. These variables, which represented up to 90% of the total data variability, were held in order to be used in the next step of the process. Also, a rotated factor pattern of the variables retained was created using the varimax method [21,22].
- *Study of selected variables similarity by means of a dendrogram*: The remaining variables were classified by means of a dendrogram in order to find those which are most similar to the output variable, which in this case is the remaining useful life.
- *Calculation of the regression tree*: Using those variables that are most similar to the output variable, a regression tree was calculated.
- *Calculation of a multivariate adaptive regression splines (MARS) model for each one of the leaf nodes of the regression tree*: A MARS model, using as input variables all those considered of relevance by the dendrogram, was calculated for each of the leaf nodes of the regression tree.

The contribution of each of the techniques included in the hybrid model is as follows: the two first methods, Principal Components Analysis and the Dendrogram are used for dimensional reduction. In the case of the PCA, it is used as a dimensional reduction tool able to eliminate those variables that contribute the least to data variance, while the dendrogram makes it possible to remove those sensors and operational settings which have a weak relationship with the RUL. The aim of the regression tree in the framework of the proposed hybrid algorithm is to create different subsets of input variables values according to their RUL. Finally, a different MARS model was trained for each of these subsets. The advantages of the proposed algorithm are twofold; on the one hand a reduced number of variables is employed for its training, which makes it more efficient, and on the other hand the final MARS models that are trained are not for the complete state of space, but for a subset which increases the accuracy of predictions over an individual method. As far as the authors know, and according to revision articles [15,16], this research presents the first application of both dendrogram and decision trees to the C-MAPSS database. The next section describes one by one all the techniques that are used in this algorithm.

Finally, the performance of the proposed hybrid model, obtained by following all the steps detailed above, was also compared with the performance obtained by two other models that have been used in recent years for the calculation of remaining useful life: multivariate linear regression [14] and back-propagation artificial neural networks [14]. In these comparisons two classical methods, as the root mean square error (RMSE) and mean absolute error (MAE), have been used because they are commonly used in literature; as the information provided by these metrics is not satisfactory for prognostics another three more suitable metrics [23] have also employed: accuracy, steadiness and risk level. Accuracy is a bias measure of the prognostic algorithm, which is defined as the relative error of the RUL prediction. The steadiness measures the volatility of the RUL prediction and is computed as the square root of the RUL predicted in function of time. In the present research it has been considered a delta of 10 time units. The smaller the value of the steadiness the more stable the predictions [24]. The risk level is the probability of obtaining a RUL estimate smaller than the true RUL.

### 2.3. Statistical Procedures Applied in the Algorithm

#### 2.3.1. Dimensional Reduction: Principal Components Analysis

The most traditional, well-known and efficient method for reducing data dimensionality by transformation is a technique called principal components analysis (PCA), which involves transforming the original attributes or variables $x_{1},x_{2},...,x_{m}$ into another set $f_{1},f_{2},...,f_{p}$, where p≤m. The PCA method was independently introduced by Pearson in 1901 [23] and Hotelling in 1933 [24]. The use of PCA as a pre-processing tool is extremely well-documented in literature and is commonly accepted. There are several recent papers that used it in this way [25,26,27]. From a geometrical point of view, this process can be seen as a change of axes in the representation (projection) [21]. From a mathematic point of view can be expressed as:

(1) $$\underset{\left(m\times 1\right)}{\bar{f}}=\underset{\left(m\times m\right)}{\left[\begin{array}{c} {\bar{a}}_{1}^{T}\\ {\bar{a}}_{2}^{T}\\ .\\ .\\ .\\ {\bar{a}}_{m}^{T} \end{array}\right]}\;\underset{\left(m\times 1\right)}{\bar{x}}=\underset{\left(m\times m\right)}{A^{T}}\underset{\left(m\times 1\right)}{\bar{x}}$$

In other words, it is necessary to get a m×m coefficients matrix which, multiplied by each vector of the original attributes, transform it into another vector in a new dimensional space. The Principal Components Analysis assumes that it is required that the variance of the new attributes be ordered according to their value, from larger to smaller. It can be demonstrated that the calculus of the result consists of only the calculus of the covariance matrix [23]:

(2) $$\underset{\left(m\times m\right)}{S}=\frac{1}{n-1}\sum_{i=1}^{n}\underset{\left(m\times 1\right)}{\left({\bar{u}}_{i}\right)}\cdot \underset{\left(1\times m\right)}{{\left({\bar{u}}_{i}\right)}^{T}}$$

where *n* is the number of examples in the data set, *m* is the number of attributes and the vectors of differences ${\bar{u}}_{i}$ are calculated subtracting each example *i* (attributes vector $x_{1},x_{2},...,x_{m}$) with the average value of all the examples [23]:

(3) $$\underset{\left(m\times 1\right)}{{\bar{u}}_{i}}=\underset{\left(m\times 1\right)}{{\bar{x}}_{i}}-\underset{\left(m\times 1\right)}{\bar{m}}\;\mathrm{where}\;\underset{\left(m\times 1\right)}{\bar{m}}=\frac{1}{n}\sum_{i=1}^{n}\underset{\left(m\times 1\right)}{{\bar{x}}_{i}}$$

After the calculus of the covariance matrix *S*, the coefficient vectors are obtained by calculating the eigenvalues of the covariance matrix:

(4) $$e_{1}\geq e_{2}\geq ...\geq e_{m}\geq 0$$

There are different criteria for determining how many dimensions to preserve. For example, in this case, as many dimensions as would be necessary to add up to at least 90% of the variance of the original data will be chosen. Another criterion, for example, chooses all those dimensions whose variance is greater than the average of the eigenvalues. In our case a quite restrictive criterion was chosen, as PCA is used as a first stage tool for pruning variables. It involved adding at least as many dimensions as would be necessary to achieve 90% of the variance of the original data.

#### 2.3.2. Dendrograms

A dendrogram is a kind of tree diagram frequently used to illustrate the arrangement of clusters obtained after a hierarchical clustering process [21]. The top row of nodes represent individual observations or data, while the remaining nodes represent the groups to which the data belongs and the arrows, the distances or differences. To perform this grouping, it is usual to use the Euclidean distance as the distance metric. In the present research the metric referred to was employed.

The Euclidean distance between two points $\left(x_{i1},x_{i2},...,x_{ip}\right)$ and $\left(x_{j1},x_{j2},...,x_{jp}\right)$ in a p dimensions space can be defined as follows:

(5) dij=(xi1−xj1)2+(xi2−xj2)2+...+(xip−xjp)2

In the case of the present research, and before the application of the Euclidean distance, variables are normalized in order to avoid problems related to their different scales.

#### 2.3.3. Decision Trees

A decision tree is a set of terms in a hierarchical structure arranged in such a way that the final decision shall be determined following the conditions that are present in each branch of the tree, from its roots to its leaves [21,28]. One of the great advantages of decision trees is that, in its general form, the possible options from a given condition are exclusive. This allows a situation to be analysed and, following the decision tree properly, action to be taken or one decision to be made. In the case of the present research, an implementation of recursive regression partitioning trees was employed [29].

#### 2.3.4. Multivariate Adaptive Regression Splines Models (MARS)

The multivariate adaptive regression models (MARS) is a technique of classification or multivariate nonparametric regression introduced by Friedman [29,30,31,32,33,34,35]. Its main purpose is to predict the values of a continuous dependent variable y(n×1) from a set of independent explanatory variables X(n×p). The MARS model can be represented as:

(6) y=f(X)+e

where *f* is the sum of the base functions and e is an error vector of dimensions (n×1).

One of the main advantages of the MARS model is that it does not require any *a priori* assumptions about the functional relationship between the dependent and independent variables. This relationship is modelled by a subset of coefficients and piecewise linear splines (basic functions). The basis functions span the space of *q*-th order spline approximation, fitting the coefficients of the basis function expansion to the data by ordinary least-squares. In the univariate case (n=1) with K+1 regions delineated by *K* points on the real line (knots), one such basis is represented by the function [29,30,31]:

(7) $${\left[-\left(x-t\right)\right]}_{+}^{q}=\{\begin{array}{cc} {\left(t-x\right)}^{q} & if\;\;x<t\\ 0 & otherwise \end{array}$$

(8) $${\left[+\left(x-t\right)\right]}_{+}^{q}=\{\begin{array}{cc} {\left(t-x\right)}^{q} & if\;\;x\geq t\\ 0 & otherwise \end{array}$$

where q(≥0) is the power of the basis functions. The MARS model of a dependent variable y with *M* basis functions (terms) can be described as follows [21,27,28]:

(9) $$\hat{y}={\hat{f}}_{M}\left(x\right)=c_{0}+\sum_{m=1}^{M}c_{m}B_{m}\left(x\right)$$

where $\hat{y}$ is the dependent variable predicted by the MARS model, $c_{0}$ is a constant, $B_{m}\left(x\right)$ is the *m*-th basis function and $c_{m}$ is the coefficient of the *m*-th basis function. Both variables entered in the model as the positions of the nodes for each individual variable must be optimized. For each data set X containing *n* objects and *p* explanatory variables, there are N=n×p pairs of basis functions given by Equations (2) and (3), with the locations of the nodes $x_{ij}$(i=1, 2,...,n;  j=1, 2,...,p). The final model is built using a two-step model. Firstly, in order to select the consecutive base pairs of the model functions, a method is implemented of two steps forward each time [29,30,31]. This progressive selection of basis functions leads to a very complex and overfitted model. Such a model, although it fits the data well, has poor predictive ability for new objects. To improve prediction, redundant base functions are removed one at a time using a regression procedure. To determine which basis functions will be included in the model, MARS uses the generalized cross validation (*GCV*) [29,30,31,32,33,34,35]. Thus, the *GCV* is the RMS residual error divided by a penalty parameter, which depends on the complexity of the model. *GCV* criterion is defined as follows [29,30,31,32,33,34,35]:

(10) $$GCV\left(M\right)=\frac{\frac{1}{n}\sum_{i=1}^{n}{\left(y_{i}-{\hat{f}}_{M}\left(x_{i}\right)\right)}^{2}}{{\left(1-C\left(M\right)/n\right)}^{2}}$$

where C(M) is the penalty due to the complexity, which increases with the number of basis functions, and the model is defined as [29,30,31]:

(11) C(M)=(M+1)+dM

where *M* is the number of basis functions in Equation (7), and the parameter d is a penalty for each base function included in the model. In the present study, the parameter *d* is equal to 2, and the maximum number of tracer interaction type base functions is restricted to 3.

## 3. Results and Discussion

This section presents the results obtained from the application of the data-driven hybrid algorithm proposed in this paper. These results are discussed and the performance compared with that obtained using different techniques for the same database. Finally, the advantages of this new algorithm are also mentioned.

The database used for evaluating the performance of the hybrid model consisted of a total of 16,609 observations, corresponding to a total of 100 different aircraft engines. Besides the identification number of the aircraft engine to which the data belongs, each observation contains a total of 25 variables: the remaining useful life, together with the values for three operation settings and a total of 21 measurements belonging to different sensors located in the aircraft engine. Table 2 summarizes the descriptive statistics of the database. In order to perform the training of all the models, information was used from 80 out of the 100 aircraft engines available, chosen at random. Afterwards, the validation of the models was made using the data from the 20 remaining aircraft engines. In other words, the engine set was randomly divided into two subsets: one with 80 engines and the other with the 20 remaining engines. The information subset with 80 engines was used for the training and the other 20 for the validation. The validation process consisted of applying the prediction model to the values of the variables in the other 20 models in order to predict their remaining useful life. This sequence of random division of the data available on the basis of training data and validation was repeated five times, and the predicted results were compared with the real values of the remaining useful life.

**Table 2:** Descriptive statistics of all the variables of the database.

Input Variables	Mean	Standard Deviation
Remaining useful life	108.808	68.881
Operational setting 1	−8.870 × 10^−6^	0.003
Operational setting 2	2.350 × 10^−6^	0.003
Operational setting 3	100.000	10^−6^
Total temperature at fan inlet (°R)	518.670	10^−6^
Total temperature at LPC outlet (°R)	642.681	0.500
Total temperature at HPC outlet (°R)	1590.523	6.131
Total temperature at LPT outlet (°R)	1408.934	9.000
Pressure at fan inlet (psia)	14.620	10^−6^
Total pressure in bypass-duct (psia)	21.609	0.001
Total pressure at HPC outlet (psia)	553.368	0.885
Physical fan speed (rpm)	2388.097	0.070
Physical core speed (rpm)	9065.243	22.082
Engine pressure ratio (P50/P2)	1.300	10^−6^
Static pressure at HPC outlet (psia)	47.5412	0.267
Ratio of fuel flow to Ps30 (pps/psi)	521.414	0.738
Corrected fan speed (rpm)	2388.096	0.719
Corrected core speed (rpm)	8143.753	19.076
Bypass ratio	8.442146	0.038
Burner fuel-air ratio	0.0300	10^−6^
Bleed enthalpy	393.212	1.549
Demanded fan speed (rpm)	2388.000	10^−6^
Demanded corrected fan speed (rpm)	100.000	10^−6^
HPT coolant bleed (lbm/s)	38.8163	0.181
LPT coolant bleed (lbm/s)	23.279	0.108

First of all, a multivariate linear regression model was performed using the remaining useful life as the output variable and the rest of the available variables as input variables. The model obtained had a $R^{2}$ value of 0.5846 (the average value of five replicates where the model was trained with 5 different training subsets). The application of this model to the multivariate linear regression validation data subset provided a result for the RMSE of 44.9307 (the average of five repetitions), a MAE of 33.6063 (the mean of the five repetitions) and an accuracy of 0.5635 (the average of five repetitions). Afterwards, a back-propagation neural network model with one hidden layer was trained, obtaining a RMSE of 42.3311 (the average of five repetitions), a MAE of 29.6364 (the mean of the five replicates) and an accuracy of 0.4996 (the average of five repetitions). The aim of the training and validation of both models was to obtain two benchmark references and compare the hybrid model presented in this research. In order to make it easy to compare the results obtained by means of the different techniques, the five training and validation subsets employed for all the models were the same.

Afterwards, the proposed hybrid algorithm was applied, producing the following results:

- *Dimensional reduction by means of principal component analysis*: The PCA algorithm was fed with 24 variables: the three operational settings and the 21 sensor measurements. A total of seven variables were discarded, and thus the total input variables subset was reduced from 24 to 17, containing a 91.2% of the variability of the data. This percentage of variability was explained by the seven *principal components* which were retained. A rotated pattern of variables was created using varimax method. Variables and factor loadings are shown in Table 3. Please note that $h^{2}$ represents the communality estimate and measures the ratio per unit of variance in an observed variable accounted for by the retained components. When the research was conducted, the use of a larger number of dimensions was checked. Using eight dimensions increased the variability contained to 92.1% and two variables more were added to the remaining variables subset, passing from 17 to 19. Nevertheless, the use of eight principal components was ruled out as in the next stage of the algorithm neither of the two new variables included were preserved when similarities were calculated by means of the dendrogram. The steps of the algorithm after the PCA are not performed in the space of the principal components as the next step of the algorithm is a dendrogram, in which similarities among variables and the RUL are analyzed; and although the model developed in this paper is data-driven, it is worthwhile to have the information corresponding to the original variables rather than the transformed ones, in order to be able to analyze the variables that take part in the model. Please also note that the variable subset obtained after this stage was also used for the training of both the linear regression and neural network models previously presented without any remarkable improvement in their results.

**Table 3:** Rotated pattern of variables with loadings and estimated communality.

Variable	PC1	PC2	PC3	PC4	PC5	PC6	PC7	$h^{2}$
Op.Set1	0.01	−0.01	1	0.01	0	0	0	1
Op.Set2	0.01	−0.01	0.01	1	0	0	0	1
Sensor.Measurement2	0.7	0.11	0.01	0	0.1	0.09	0.69	0.99
Sensor.Measurement3	0.63	0.16	0	0	0.75	0.09	0.09	1
Sensor.Measurement4	0.87	0.12	0.01	0.01	0.14	0.14	0.12	0.82
Sensor.Measurement7	−0.87	−0.02	0	−0.01	−0.14	−0.14	−0.12	0.81
Sensor.Measurement9	0.19	0.97	0	0	0.06	0.06	0.04	0.98
Sensor.Measurement11	0.89	0.08	0.01	0	0.14	0.14	0.14	0.86
Sensor.Measurement12	−0.89	−0.01	0	0	−0.15	−0.14	−0.14	0.85
Sensor.Measurement14	0.07	0.99	0	0	0.04	0.04	0.02	0.98
Sensor.Measurement15	0.85	0.13	0	0.01	0.09	0.09	0.08	0.76
Sensor.Measurement17	0.67	0.17	0	0	0.1	0.71	0.09	0.99
Sensor.Measurement20	−0.88	−0.13	0	0	−0.05	−0.01	0	0.79
Sensor.Measurement21	−0.85	−0.13	−0.01	0	−0.09	−0.05	−0.05	0.76

**Figure 3:**
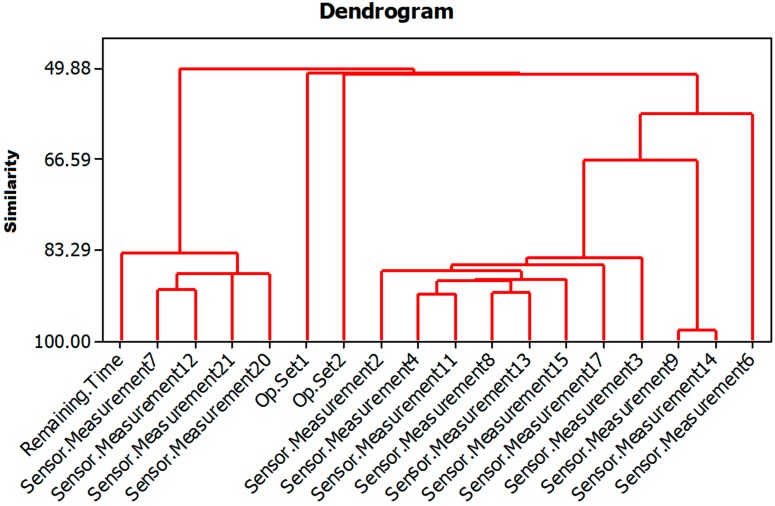
Dendrogram of the remaining useful life variable and the other variables after the dimensional reduction.

**Figure 4:**
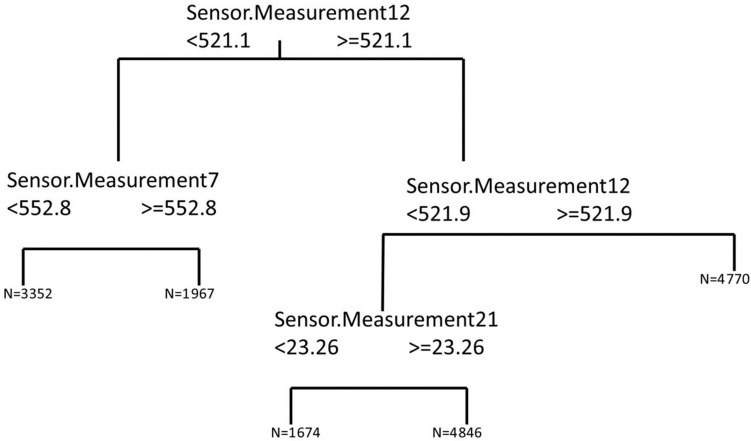
Regression tree of the remaining useful life variable using as input variables Sensor.Measurement7, Sensor.Measurement12, Sensor.Measurement20 and Sensor.Measurement21.

- *Making a dendrogram with the selected variables*: Figure 3 shows the dendrogram obtained with the 17 variables selected by the PCA plus the RUL. In this dendrogram, what can clearly be seen is the degree of similarity between variables, which will be decisive in choosing those variables that are related to the remaining useful lifetime (RUL) variable. The dendrogram shown in Figure 3 corresponds to one of the five repetitions. All the dendrograms obtained were very similar. The variables selected corresponded to four sensors: Sensor.Measurement7, Sensor.Measurement12, Sensor.Measurement20 and Sensor.Measurement21. As can be observed in Figure 3, these four variables have the largest degree of similarity to the remaining useful life variable. Therefore, in this step of the algorithm the number of input variables was reduced from 18 (17 plus the RUL) to 4.

- *Calculating a regression tree using variables that are more similar to the variable remaining useful* *life*: For this problem, the four variables in the dendrogram that showed a level of similarity to the remaining useful life (called Remaining.Time in Figure 3) over 83.29% were used as input variables for a regression tree in which the remaining useful life is the output variable. The regression tree obtained is shown in Figure 3, and used to determine the cut-off points corresponding to the input variables. As can be observed in Figure 4, although four input variables were proposed for the regression tree model, only three are used for the cut-off points. The variables referred to are the following: Sensor.Measurement7, Sensor.Measurement12 and Sensor.Measurement21. The numbers named as N in the leaf nodes of the regression tree represent the number of cases that are in said node and that will therefore be used for the training of the corresponding MARS model.
- *Training a MARS model*: after the performance of the regression tree, a total of five different MARS models were trained. Each of these models corresponded to one of the end nodes of the regression tree. Each of the MARS models was trained with the part of the training subset that satisfied the conditions of their corresponding branches of the tree, using as dependent variables the four obtained as the outcome of the dendrogram (Sensor.Measurement7, Sensor.Measurement12, Sensor.Measurement12 and Sensor.Measurement21).

**Figure 5:**
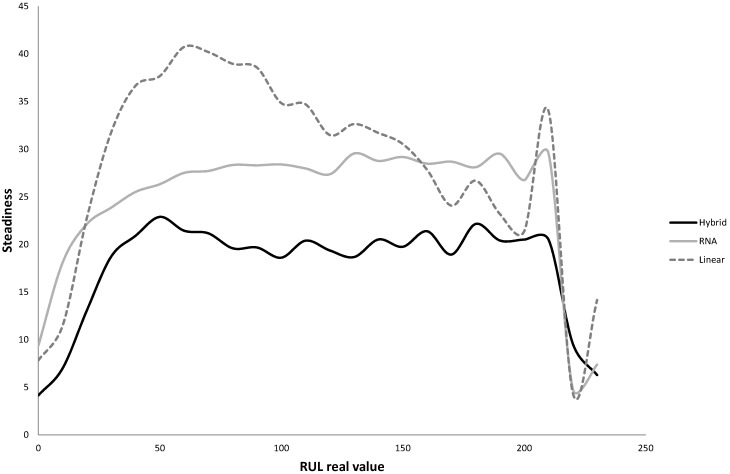
Steadiness of the hybrid model compared with the two benchmark techniques.

**Figure 6:**
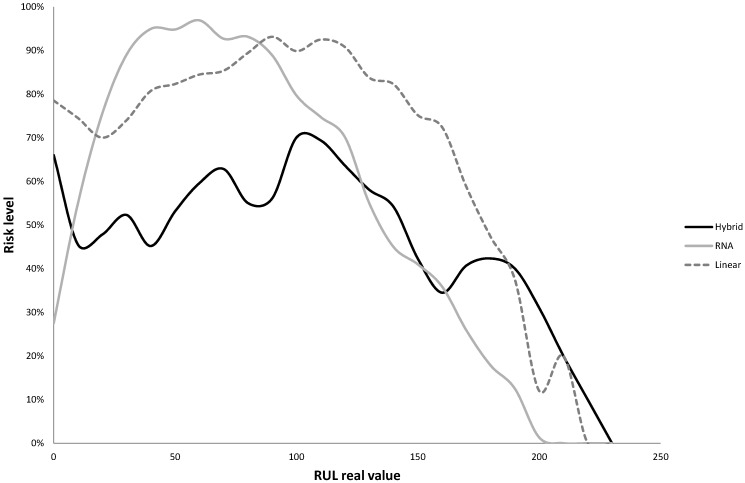
Risk level of the hybrid model compared with the two benchmark techniques.

After the training of the MARS models referred to in the last stage of the algorithm, the resulting hybrid model was applied to the validation data subset, resulting in RMSE and MAE values of 36.0836 and 21.5624 respectively (the mean values of five replications of the algorithm), with an average value for the accuracy of 0.3123 (five replications). The values of steadiness and risk levels were also calculated for the hybrid and the two benchmark models. The results of the steadiness are presented in Figure 5. As can be observed the steadiness of the hybrid model is better than that obtained by both the linear and RNA models for almost any of the RUL values considered. Figure 6 shows the risk level of each model. In this case, the probability of obtaining a RUL estimate smaller than the true RUL is higher with the linear and hybrid models than with the hybrid one. In spite of this result, which would seem to be a better performance from both linear and hybrid models, we would like to remark that the predictions of both models are quite far from the real RUL when compared with the hybrid model. In other words, both models underestimate the RUL, but this underestimation is too far from the real value to be useful.

According to the results of the regression tree, the three most relevant variables for the determination of the RUL of the aircraft engine are Sensor.Measurement7 (total pressure of the HPC outlet), Sensor.Measurement12 (ratio of fuel flow to PS30) and Sensor.Measurement21 (LPT coolant bleed). The turbine compressor is required to have a constant outlet pressure despite the fluctuating turbine load and fluctuating supply. According to the results, variations in the total pressure of the HPC outlet are important in order to predict the engine’s RUL. The presence of the ratio of fuel low to PS30 as one of the remaining input variables allows us to connect the RUL of the aircraft engines with this variable and also indirectly with the gross shaft power of the engine and the possible power loss that would be found due to degradation during the engine operation. 

**Figure 7:**
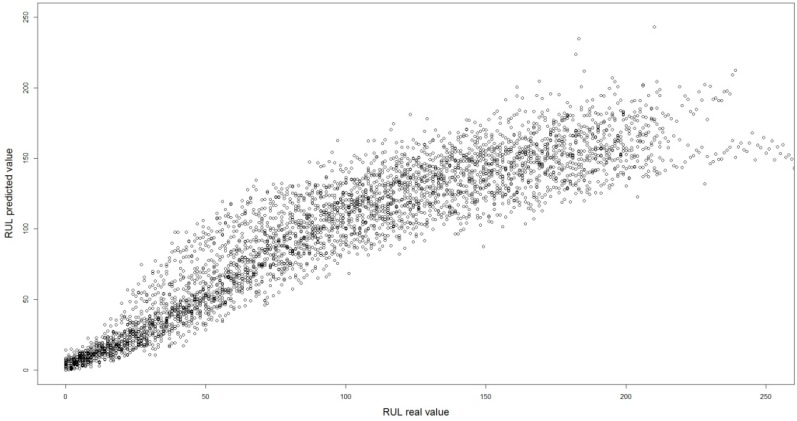
Remaining useful life of one of the validation subsets *versus* the remaining useful life calculated by the hybrid model.

From a design point of view, the geometry of a coolant channel of a LP blade is relatively simple in comparison with that of HP blade in spite of this, flow variations in during the operation of the aircraft engine, have been shown to be linked to a decrease in the RUL of the aircraft engine. The results obtained in the five replications were statistically equivalent when compared by means of a one-way ANOVA test. The range of dispersion of differences between real and predicted values changes significantly from one aircraft engine to another. The same pattern of behavior was found in the regression and neural network models. In spite of this, the RMSE results obtained by means of the artificial neural network was 14.76% greater than those obtained by means of the hybrid model. In the case of the MAE differences the figure was 27.24% more than with the hybrid method. Figure 7 shows the remaining useful life of one of the validation subsets *versus* the remaining useful life calculated by means of the hybrid model. As may be observed, there is a visual correlation that corresponds to a Pearson’s correlation coefficient of 0.9167.

Table 4 shows the confusion matrix of the hybrid, multivariate linear regression and back-propagation neural network models. As the RUL is a continuous variable, a cut-off point was defined in order to determine which aircraft engines were close to the end of their useful life. As it was not possible to establish objective engineering criteria, it was considered that those situations in which the actual RUL of the engine was below 15% of the maximum RUL value considered in the study (312 time units) meant that the unit was close to the end of its useful life. According to the values of this table, the overall misclassification rate of the hybrid model is 5.72%, while the rate for the linear regression and neural networks models were respectively 8.43% and 6.91%. In the case of sensitivity, the highest value was achieved by the hybrid model with a 92.20%, followed by the neural network model with 82.55% and the linear regression model with 65.64%. Finally, in the case of the specificity, the highest value is for the linear regression model with 99.67%, followed by the neural network with 96.38% and the hybrid model with 94.85%. Please note that in this problem the most important parameter is the sensitivity, as it shows the ability of the model to detect engines close to the end of their useful life. Finally, and due to the arbitrary selection of the cut-off point, we would like also to point out that in the case of having considered a cut-off point corresponding to 10% of the maximum remaining useful life of the validation data subset, the sensitivity and specificity values of the hybrid model would have been 86.57% and 97.23% respectively and in the case of the 20% they would be 94.81% for sensitivity and 91.19% for specificity.

**Table 4:** Confusion matrix of the hybrid, multivariate linear regression and back-propagation neural network models.

		Hybrid		Linear Regression		Neural Network	
	Predicted						
		Negative	Positive	Negative	Positive	Negative	Positive
**Actual**	**Negative**	2946	66	3002	10	2903	109
	**Positive**	160	780	323	617	164	776

According to the report published by Ramasso and Saxena [36] in 2014, up to that time more than seventy publications had developed prognostic algorithms using the C-MAPSS datasets. The paper reported the 10 best models, from the authors’ point of view that had been applied to the C-MAPSS data sets. Three of them correspond to an unpublished master’s thesis and two unpublished doctoral dissertations. When compared with those models, the one presented in the current research outperformed the correlation score of 7 out of 10 [37,38,39,40,41,42,43] and the MAE of 3 out of 10 [41,42,43]. Please note that on the one hand the training and validation sets used in the different articles are heterogeneous and that on the other hand, no detailed information of their results is available (real RUL values *versus* estimated RUL values for all the referred works). It must also be taken into account that MAE results should be treated with care, as the reliability of this parameter is partially linked to the sample size [44] which varies from one research to another. In the case of the three studies that would appear to outperform the result obtained in this research, the solutions proposed by Heimes [45] and Peel [46] require the use of different multilayer perceptron neural networks, with both models using the 24 variables as inputs for the model. In the case of the research by Heimes, the multilayer perceptron neural network has 24 neurons in the input layer and two hidden layer, one with three neurons and another with two. This model requires a minimum RUL length of 127 cycles. Such a demanding requirement concerning RUL length is not necessary for our model. In the case of the model proposed by Peels, not only did it use 24 input variables, but it was also an ensemble model, composed of two multilayer perceptron neural networks and a Kalman filter. Finally, the approach of Wang [14] is more similar to our research, but requires a total of 7 input variables. 

As may be noticed, the algorithm proposed in the present paper performs a dimensional reduction by means of PCA, and afterwards a study of the similarity of the remaining variables with the output variable Remaining Time is performed. Despite the similarity of both methods, previous research [22,47] has demonstrated that the use of both techniques together, PCA with varimax rotation and hierarchical clustering by means of a dendrogram, are more effective than the use of either one of them alone. This fact is confirmed by the results of the present papers although no comparison with the use of only one of these techniques is presented as it is not the main purpose of this research. Similar methodologies have also been used in other research [48] with satisfactory results.

Finally, from the authors’ point of view, it would be also worth highlighting that although it is possible to find in the literature better results for predictions performed over the same database, the main advantage of the present approach is that it does not require the use of the previous engine states unlike in other models [49], which in some cases makes it necessary to have a record length of even 500 consecutive data [50] in order to detect the fault with a reasonable confidence. In other words, the output variable, the RUL, is calculated at any given moment knowing only the values of the input variables in the same moment. Therefore it is not necessary to store previous values of these variables. Of course, the value of any input variable at any given moment would be conditioned by the previous operational conditions and degradation status of the engine, but such information is not directly required as a direct input for the model.

## 4. Conclusions

In this paper, a hybrid prognostics approach which integrates the MARS method and PCA plus CART techniques is presented. It is a well-known fact that fault prognosis is an essential and complex technology within health management and CBM. Testing of the proposed MARS-PCA-CART-based prognostic approach was carried out using sensor data from aircraft engines. The simulation results showed that the new approach is efficient in predicting the remaining useful life (RUL) for the aircraft engines, as it improved the results of the benchmark technique. The proposed hybrid model improves upon the performance of classical predictive models used for comparison (linear multivariate regression model and neural network model with back propagation) [12,51,52,53,54]. It has also been stated in literature on predictive maintenance that one of the most important issues in prognostic technology [55] is the estimation of the risk of failure and the RUL of a component, given the component’s age and its past and current operating conditions. In the case of the model proposed in this paper, when used for the prediction of the RUL, it does not require information about the previous status of the variables of the engine: it only requires information regarding the current situation of said variables. Please note that this does not mean that the model is not affected by the previous conditions of the engine, but that the only input variables required for the prediction belong to the current temporal moment and not to any previous status. However, of course, the values of the input variables at each temporal moment will certainly be affected by the previous operative conditions of the engine, and also by the previous engine status. In other words, this model provides two main advantages: firstly, system robustness against possible failures in the memory registers, as well as a reduction in the complexity of actual implementation, which is linked to fewer variables to monitor in real time. Please note that in the end, the number of variables required for the prediction of remaining useful life is a total of four. In other words, the algorithm presented in this paper is suitable for its application to any other system we have historical records for regarding its variables, and where the information about the RUL for each data set is available. This algorithm is particularly recommended if the initial number of variables implied is large enough to require a previous dimensional reduction and there are non-linear relationships of the input variables with the RUL. According to the research of Sikorska *et al.* [16], the model developed in the present research would be considered as a Level 1 model in the prognostics category.

As a future line of research, the authors propose the performance of models taking into account the values of the input variables at earlier time points, in order to determine the remaining useful life of the aircraft engines. From the authors’ point of view, these models should provide a better fit, but suffer from the need to have in memory the values at previous time points of the variables included in the model. These models would be of interest only if they could be trained with fewer variables than the model that does not require information of the previous state. Finally, this future line of research should be completed with a test on real data as the simulations performed in this research were made on simulation data.
